# No significant change of N^6^‐methyladenosine modification landscape in mouse brain after morphine exposure

**DOI:** 10.1002/brb3.3350

**Published:** 2023-12-31

**Authors:** Xiaoli Wu, Cuiting Wu, Tao Zhou

**Affiliations:** ^1^ Shenzhen Neher Neural Plasticity Laboratory, Shenzhen Key Laboratory of Drug Addiction, the Brain Cognition and Brain Disease Institute, Shenzhen Institute of Advanced Technology Chinese Academy of Sciences Shenzhen China; ^2^ University of Chinese Academy of Sciences Beijing China; ^3^ Shenzhen‐Hong Kong Institute of Brain Science‐Shenzhen Fundamental Research Institutions Shenzhen China; ^4^ CAS Key Laboratory of Brain Connectome and Manipulation, Faculty of Life and Health Sciences, the Brain Cognition and Brain Disease Institute, Shenzhen Institute of Advanced Technology Chinese Academy of Sciences Shenzhen China

**Keywords:** epigenetics, morphine, N6‐methyladenosine, opioid, posttranscriptional RNA modification, reward

## Abstract

**Objectives:**

N^6^‐methyladenosine (m^6^A) plays a crucial role in regulating neuroplasticity and different brain functions at the posttranscriptional level. However, it remains unknown whether m^6^A modification is involved in acute and chronic morphine exposure.

**Materials and methods:**

In this study, we conducted a direct comparison of m^6^A levels and mRNA expression of m^6^A‐associated factors between morphine‐treated and nontreated C57BL/6 wild‐type mice. We established animal models of both acute and chronic morphine treatment and confirmed the rewarding effects of chronic morphine treatment using the conditioned place preference (CPP) assay. The activation status of different brain regions in response to morphine was assessed by c‐fos staining. To assess overall m^6^A modification levels, we employed the m^6^A dot blot assay, while mRNA levels of m^6^A‐associated proteins were measured using a quantitative polymerase chain reaction (qPCR) assay. These analyses were performed to investigate whether and how m^6^A modification and m^6^A‐associated protein expression will change following morphine exposure.

**Results:**

The overall m^6^A methylation and mRNA levels of m^6^A‐associated proteins were not significantly altered in brain regions that were either activated or not activated during acute morphine stimulation. Similarly, the overall m^6^A modification and mRNA levels of m^6^A‐associated proteins remained unaffected in several key brain regions associated with reward following chronic morphine exposure.

**Conclusion:**

This study showed that the overall m^6^A modification level and mRNA expression levels of m^6^A‐associated factors were not affected after acute and chronic morphine exposure in different brain regions, indicating m^6^A modification may not be involved in brain response to morphine exposure.

## Graphical abstract

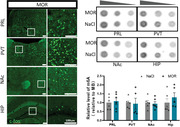

## 1 INTRODUCTION

In addition to the abuse of recreational stimulants, frequent use of analgesics in the clinic, such as morphine, also eventually leads to a high rate of abuse potential. The prolonged use of opioid drugs with abuse potential can result in physical or psychological dependence, contributing to a global public health crisis. The formation of opioid dependence is dependent on the rewarding effect of drugs and is closely related to neural adaptation in the central nervous system at the molecular and cellular levels (Koob et al., 2004; Motahari et al., 2016; Nestler, 2004; Robinson & Kolb, 2004; Spanagel & Shippenberg, 1993; Spanagel et al., 1993; Tjon et al., 1994, 1997). However, the precise molecular mechanisms behind these changes remain to be fully elucidated. In recent years, increasing attention has been paid to the role of epigenetic modifications in opioid exposure, including DNA methylation and chromatin modifications such as histone modification (Barrow et al., 2017; Chorbov et al., 2011; Doehring et al., 2013; Hwang et al., 2007; Mashayekhi et al., 2012; Wei et al., 2016). Repeated exposure to opioids causes changes in the epigenetic landscape of brain cells, leading to persistent alterations in transcriptional activity and cell physiology (Bazov et al., 2018; Hwang et al., 2007). In sensory neurons, cell‐specific alternative splicing of the presynaptic Ca**_V_** channel Cacna1b gene has been found to modulate opioid sensitivity (Lopez Soto & Lipscombe, 2020). Moreover, epigenetic upregulation of postsynaptic density protein 95 (PSD‐95), mediated by cAMP response element‐binding protein (CREB), contributes to morphine‐induced reward behavior (Wang et al., 2014). And long‐term exposure to morphine impairs the ability of histone methyltransferase‐G9a to regulate abnormal gene expression in the nucleus accumbens (NAc) (Wang et al., 2014).

Similar to DNA modifications, RNA also undergoes multiple covalent epigenetic modifications. m^6^A is the most prevalent mRNA modification in mammals and is catalyzed by methyltransferase complexes, which include methyltransferase‐like 3 (METTL3), methyltransferase‐like 14 (METTL14), vir‐like m^6^A methyltransferase‐associated protein (VIRMA), Wilms tumor 1‐associated protein (WTAP), zinc finger CCCH domain‐containing protein 13 (Zc3h13), and Casitas B‐lineage lymphoma‐transforming sequence‐like protein 1 (CBLL1) (Bokar et al., 1997; Knuckles et al., 2018; Su et al., 2022; Yue et al., 2018). m^6^A can be specifically removed by the fat mass and obesity‐associated protein (FTO)/ alkB homolog 5 (ALKBH5) (Jia et al., 2011; Zheng et al., 2013). After m^6^A modification, recognition proteins such as YTH‐domain‐containing proteins (YTHDF1/2/3) can bind to it, leading to regulation of RNA maturation, splicing, alternative polyadenylation, RNA decay, and translation, ultimately influencing gene expression. m^6^A is highly abundant in the brain (Meyer et al., 2012). Many studies have demonstrated the significant role of m^6^A in brain development, learning, memory, synaptic plasticity, and stress response in the nervous system (Leonetti et al., 2020; Lv et al., 2022; Pupak et al., 2022; Shi et al., 2018; Widagdo et al., 2022). Whereas, relatively few studies investigated the role of m^6^A modifications in addictive drug exposure, especially in the context of opioids like morphine. The regulatory effects of FTO and YTHDF1 on addictive drugs suggest that m^6^A might play functional roles in drug induced reward‐related behaviors. For example, dysregulation of m^6^A epitranscriptomic and FTO expression in the hippocampus following cocaine‐induced CPP (Xue et al., 2021), FTO is present in dopamine neurons and controls cocaine responses (Hess et al., 2013), YTHDF1‐TRAF6 pathway regulates the neuroinflammatory response and contributes to morphine tolerance (Ouyang et al., 2022). These findings warrant further investigation to better understand the molecular mechanisms underlying morphine exposure and to explore potential therapeutic targets.

In this study, by comparing the m^6^A modifications and m^6^A‐associated factors expression in different brain regions under acute and chronic morphine treatment, we characterized the overall m^6^A landscape in morphine‐responsive and reward‐related brain regions. Generally, both m^6^A modifications and m^6^A‐associated gene expression were not significantly changed after acute or chronic morphine stimulation, indicating that m^6^A may not be the main epigenetic regulator involved in morphine exposure.

## 2 METHOD

### 2.1 Animals and reagents

Male C57BL/6j mice aged 8−12 weeks, used in the experiments, were obtained from Charles River Laboratories in Zhejiang. All animal procedures were conducted in accordance with animal care guidelines approved by the Institutional Animal Care and Use Committee of Shenzhen Institute of Advanced Technology, Chinese Academy of Sciences. The animals were maintained under a regular 12‐h light‐dark cycle with lights on at 08:00, at a temperature of 21 ± 1°C, and in an environment with 50 ± 5% humidity. They had free access to water and food. Morphine was purchased from China National Accord Medicines.

### 2.2 Acute morphine treatments

Male C57BL/6j mice were initially acclimated to the testing environment for 3 days. Subsequently, they received a single intraperitoneal injection of either morphine (20 mg/kg; Northeast Pharmaceutical Group Shenyang First Pharmaceutical Co., LTD) or an equal volume of 0.9% NaCl. The dosage of acute morphine was referred to reported studies (Almeida‐Santos et al., 2014; Marinho et al., 2015; Wang et al., 2009). This was conducted within a two‐compartment CPP apparatus with dimensions of 30 cm×25 cm×20 cm. The left side was marked with a diamond pattern and a smooth floor, the right side was marked with stripes and a rough floor, separated by a movable partition. Tissue samples (collected from prelimbic cortex (PRL), paraventricular thalamic nucleus (PVT), nucleus accumbens (NAc), secondary motor cortex (M2), and hippocampus (HIP)) were obtained 90 min after the acute administration of morphine or 0.9%NaCl. The tissues were rapidly frozen in liquid nitrogen and stored at −80°C for subsequent RNA extraction (MOR, *n* = 6; NaCl, *n* = 6).

### 2.3 c‐fos histology

For c‐fos histology analysis, mice received a single intraperitoneal injection of morphine (MOR, *n* = 3–6) or 0.9% NaCl (NaCl, *n* = 3–6) in the CPP apparatus. Ninety minutes later, they were immediately anesthetized using 50 mg/kg sodium pentobarbital and perfused with 20 mL of PBS, followed by 20 mL of 4% paraformaldehyde (PFA) in PBS. The fixed tissue was then dehydrated using 30% sucrose for a duration of 48 h. A serial of 40 μm coronal brain sections was obtained using a cryotome (Leica, CM1950) and stored at −20°C for subsequent histological analysis. Before c‐fos staining, brain slices were washed three times for 10 min each with PBS. They were then blocked with 5% goat serum in PBST (PBS with 0.3% Triton X‐100) at room temperature for 2 h. Subsequently, the slices were incubated overnight at 4°C with the primary c‐fos antibody (Synaptic Systems, Cat# 226003) at a dilution of 1:4000. After washing off excess primary antibody with PBST, the slices were incubated with a secondary antibody (goat anti‐rabbit Alexa Fluor 488, Invitrogen, Cat# A11008) and Hoechst dye for nuclear staining at room temperature for approximately 2 h. Fluorescence images were captured using an Olympus VS120 Virtual Slide microscope, and the number of c‐fos positive cells was quantified using Image J.

For the quantitative analysis of c‐fos histology, brain slices were selected with a standard interval of approximately 8 slices (8 × 40 μm = 320 μm) between each selected slice. The number of slices (3–5 slices) chosen varied depending on the size of the brain region. Each point on the c‐fos statistics graph represents data from an individual animal. This data point is obtained by counting c‐fos positive cells in 3–5 brain slices from that animal and calculating the average.

### 2.4 Hot plate test

To evaluate the analgesic effect of the administered dose of morphine, we conducted a hot plate test to assess pain sensitivity. Referring to relevant research reports, the hot plate test (52.5°C, BIOSEB Dynamic Cold/Hot Plate, BIO‐CHP‐ER) was conducted only once 30 min after the injection of 20 mg/kg morphine or saline. The experimental procedure was as follows:

Prior to the experiment, the animals were acclimated to the test environment by placing them in the experimental room for three consecutive days (1 h per day). During this acclimatization period, each animal was gently handled for 3 min daily to familiarize them with the experimenter. 16 male C57BL/j mice (9 weeks old) were randomly divided into two groups: the morphine group (MOR, *n* = 8) and the saline group (NaCl, *n* = 8). On the day of the hot plate test, each animal was acclimated for 10 min in the unheated hot plate. After adapting to the environment, the animals were injected with morphine or normal saline, and hot plate detection was performed 30 min after injection. During the hot plate test, each animal was placed on the hot plate, and the following responses—paw lift, paw shake, paw lick, or jumping—were observed and recorded. A cut‐off time of 60 s was set to prevent injury. If an animal exhibited continuous paw licking or jumping, it was removed from the hot plate prematurely, and the entire process was recorded on video. The latency (in seconds) to the initial paw lick, paw lift, or paw shake was measured. Each point in the bar graph represents an individual animal.

### 2.5 Chronic morphine treatments and behavior tests

Prior to the experiment, all animals were acclimated to the test environment by placing them in the experimental room for three consecutive days (about 1 h per day). During this 3‐day acclimatization period, each animal was gently handled for 3 min daily to familiarize them with the experimenter. Throughout the experiment, the animals were transferred from their feeding environment to the experimental environment for adaptation 1 h before each experiment. On day 1 (Baseline), the mice were randomly divided into two groups: morphine group (MOR, *n* = 9) and saline group (NaCl, *n* = 9). They were placed in the center of the CPP chamber without partitions and allowed to freely explore both sides of the CPP chamber. A 15‐min activity track recording was conducted to establish the baseline place preference for each mouse. The preferred side in the CPP chamber of the animal was named A box and the unpreferred side was named B box. If a mouse spent more than 70 % of its time on any one side of the chamber during the baseline period, it was excluded from further behavioral assays. During days 2–5 (Training), at this stage, A box and B box of the CPP chamber were completely separated by the partition. The mice from the MOR and NaCl groups were intraperitoneally injected with 0.9 % saline on their preferred side (A box) and confined for 45 min, then returned to the home cage. At least 4 h later, the mice were intraperitoneally injected with 15 mg/kg morphine (MOR group) or an equal volume of saline (NaCl group) on their unpreferred side (B box), again confined for 45 min. This operation was performed for 4 consecutive days, ensuring that each mouse was injected with the appropriate drug and placed in the appropriate box at the same time every day. On day 6 (Test), 24 h after the final training session, the mice were placed in the center of the CPP chamber without partitions and allowed to freely explore both sides of the CPP chamber, just like during the baseline. A 15‐min video recording captured their locomotor behaviors for subsequent analysis of conditioned place preference. After completing the final test, brain regions from the mice were immediately collected and frozen at −80°C for subsequent RNA extraction.

The CPP score was calculated by subtracting the time spent on the unpreferred side (where the MOR group received morphine injections, also known as B box) during the baseline (day 1) from the time spent on the same side during the test (day 6). This is simply shown in the formula: CPP score = T6 (Time in B box of day 6) – T1 (Time in B box of day 1).

### 2.6 RNA extraction and quantitative polymerase chain reaction (qPCR)

Total RNA was extracted from various brain regions (PRL, PVT, NAc, HIP, and ventral tegmental area (VTA)) using TRIzol® Reagent (Invitrogen, 15596018), and the procedures were conducted following the manufacturer's instructions. The reverse transcription into cDNA libraries was carried out according to the protocol of the cDNA synthesis kit (Vazyme, Cat# R212‐02), and quantitative polymerase chain reaction (qPCR) was conducted using Universal SYBR qPCR master mix (Vazyme, Cat# Q511‐02). The ratio of mRNA expression was determined using the 2^−ΔΔCT^ method. The specific primer sequences used for qPCR were as follows:

ALKBH5‐F: ACA AGA TTA GAT GCA CCG CG, ALKBH5‐R: TGT CCA TTT CCA GGA TCC GG. FTO‐F: CTG AGG AAG GAG TGG CAT G, FTO‐R: TCT CCA CCT AAG ACT TGT GC. VIRMA‐F: CA TTA CGG CCG CTT AGT TCT, VIRMA‐R: TAC CAC TGC CTC CAC TAA CA. YTHDF1‐F: CAT TAT GAG AAG CGC CAG GA, YTHDF1‐R: AGA TGC AAC AAT CAA CCC CG. YTHDF2‐F: ACC AAC TCT AGG GAC ACT CA, YTHDF2‐R: GGA TAA GGA GAT GCA ACC GT. YTHDF3‐F: TGC ACA TTA TGA AAA GCG TCA, YTHDF3‐R: AGA TGC GCT GAT GAA AAC CA. GAPDH‐F: AAC GAC CCC TTC ATT GAC, GAPDH‐R: TCC ACG ACA TAC TCA GCA C. Mettl3‐F: ATT GAG AGA CTG TCC CCT GG, Mettl3‐R: AGC TTT GTA AGG AAG TGC GT. Mettl14‐F: AGA CGC CTT CAT CTC TTT GG, Mettl14‐R: AGC CTC TCG ATT TCC TCT GT. Oprm1‐F: TGC ACC ATG AGT GTA GAC CG, Oprm1‐R: GGC AGA CCA ATG GCA GAA GA.

### 2.7 m^6^A dot blot assay

The quantified total RNA samples were combined with twice the amount of incubation solution. For each milliliter of incubation solution included 210 μL of 37% formaldehyde solution, 657 μL of formamide solution, and 133 μL of 10 × MOPS. The mixture was then denatured at 65°C for 5 min, followed by immediate placement on ice to halt the reaction. Subsequently, 1 μL of denatured total RNA was placed onto a nylon membrane (Invitrogen, Cat# AM10100). After allowing it to dry at room temperature, the RNA was cross‐linked to the membrane using UV light at 254 nm. The nylon membrane was washed with TBST (0.1 % Tween‐20 in 1×TBS) and then incubated in a blocking buffer (0.5 % nonfat milk in TBST) for 2 h at room temperature. The membrane was kept overnight with anti‐m^6^A antibody (Synaptic Systems, Cat# 202003) diluted at 1:1400 in blocking buffer. Excess antibody was removed through washing with TBST. This was followed by incubation with HRP‐conjugated affinipure IgG antibody (Goat anti‐rabbit) for 2 h at room temperature. The m^6^A dot blot was visualized using a chemiluminescence reagent and counterstained with 0.02 % methylene blue to serve as the loading control. The entire process was conducted in an RNase‐free environment.

### 2.8 Statistical analyses

GraphPad Prism software version 8.0.1 (GraphPad, CA, USA) was used for statistical analysis of all data. qPCR assays were performed in technical duplicates at least two times, and m^6^A dot blot assays were performed in technical duplicates at least three times. Unpaired and multiple *t*‐tests, as well as analysis of variance (ANOVA), were employed for statistical testing. The significance level was set at 0.01 < *p < .05, 0.001 < **p < .01, and ***p < .001. Data are presented as the mean ± the standard error of the mean (SEM).

## 3 RESULTS

### 3.1 Different brain regions activated by acute morphine stimulation

**FIGURE 1:**
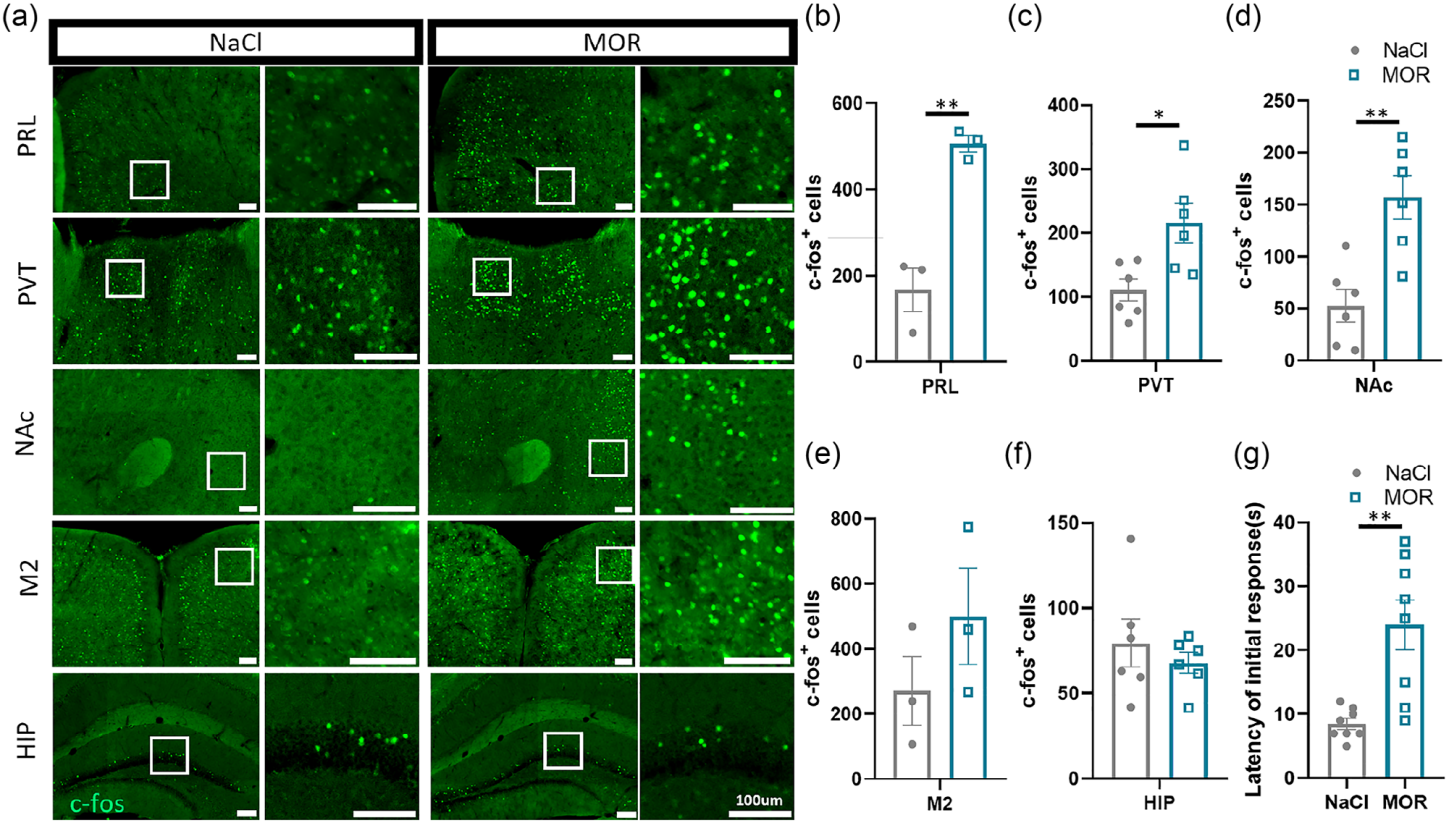
Activation of brain region following a single exposure to morphine. (a) C‐fos positive cells (green) were observed in the PRL, PVT, NAc, M2, and HIP after a single exposure to morphine or NaCl (*n* = 3–6). The square diagram on the right provides a magnified view of the field of view inside the white box on the left. Scale bar, 100 μm. PRL, prelimbic cortex; PVT, paraventricular thalamic nucleus; NAc, nucleus accumbens; M2, secondary motor cortex; HIP, hippocampus. **(b–f)** Quantification of the number of c‐fos positive cells in the PRL, PVT, NAc, M2, and HIP of mice exposed to a single dose of morphine (green) compared to control mice (gray). (*p* = .0032 in PRL; *p* = .0134 in PVT; *p* = .0026 NAc).*p < .05, **p < .01. Unpaired *t*‐test. Data represent means ± SEM (*n* = 3–6 animals.) **(g)** Hot plate test reveals a significantly longer latency of initial reaction (paw lick, paw lift or paw shake) in mice exposed to a single dose of morphine (green) compared to control mice (gray). **p < .01, Unpaired *t*‐test. Data represent means ± SEM (*n* = 8 animals).

Previous studies suggest more robust opioid analgesic responses in male relative to female animals, thus we primarily chose male mice for investigation in our study (Fillingim & Gear, 2004). In order to investigate the impact of acute morphine stimulation on m^6^A modification in different brain regions, we first conducted a comparative analysis based on c‐fos staining, which is an immediate early gene and a well‐established marker for neuronal activity, across different brain regions. The most commonly used analgesic dose of morphine ranged from 10 to 20 mg/kg in mice (Almeida‐Santos et al., 2014; Marinho et al., 2015; Wang et al., 2009), here we chose the dosage of 20 mg/kg to ensure that most animals responded to morphine. The analgesic effect of morphine was confirmed with hot plate test. The results showed that the latency of initial reaction (paw lick, paw lift, or paw shake) in morphine group (MOR, *n* = 8) was significantly longer than that in control group (NaCl, *n* = 8) (Figure 1g). At the same time, we observed that the animals in the saline group all showed hind‐paw lick and some of them showed violent jumping reaction (data not shown), while neither of these two behaviors was observed in the morphine group. These results suggest that acute morphine administration (20 mg/kg) had an obvious analgesic effect in mice. We then examined the neuronal activity in the paraventricular thalamic nucleus (PVT), prelimbic cortex (PRL), nucleus accumbens (NAc), hippocampus (HIP), and secondary motor cortex (M2) in mice following a single intraperitoneal dose of morphine (MOR, *n* = 3–6) or NaCl (NaCl, *n* = 3–6). Our findings demonstrated increased c‐fos expression in PRL, PVT, and NAc, whereas HIP did not exhibit a significant increase (Figure 1a–f). Besides, M2 showed a trend but not significantly increased c‐fos expression. These results align with previous studies (Brynildsen et al., 2020a; Jiang et al., 2021; McDevitt & Graziane, 2019). By evaluating c‐fos activation, we identified key brain regions that displayed either heightened sensitivity or negligible activation in response to a single dose of morphine. We then decided to focus on these acute morphine‐activated brain regions for further investigation.

### 3.2 m^6^A level and m^6^A‐associated factors expression in brain regions activated by acute morphine stimulation

**FIGURE 2:**
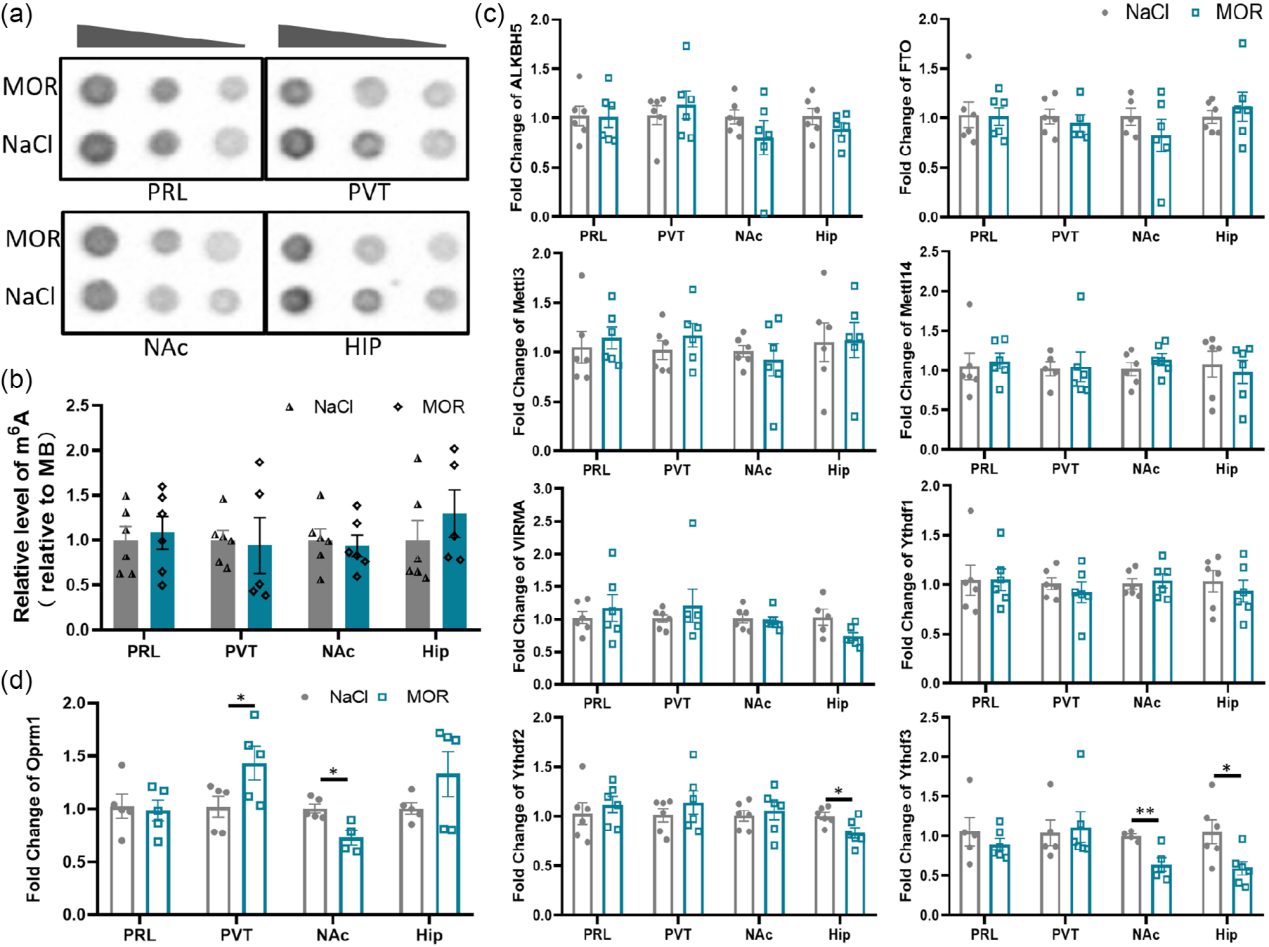
m^6^A modification landscape after single exposure to morphine. (a) The m^6^A level in total RNA was assessed using m^6^A dot blot analysis in the PRL, PVT, NAc, and HIP regions of mice exposed to a single dose of morphine‐treated (MOR, *n* = 5–6) and nontreated (NaCl, *n* = 5–6) mice. RNA samples were diluted to 150 ng, 75 ng, and 37.5 ng for the analysis. **(b)** Quantification of m^6^A dot blot in the PRL, PVT, NAc, and HIP (*n* = 5–6), with methylene blue staining serving as the loading control. (*p* = .7311 in PRL; *p* = .8528 in PVT; *p* = .7254 in NAc; *p* = .4039 in HIP), *p* ≥ .05, not significant. Multiple *t*‐test. Data represent means ± SEM (*n* = 5–6 animals.) **(c)** The relative mRNA expression levels of m^6^A‐related proteins, normalized to GAPDH as a reference, including ALKBH5, alkB homolog 5; FTO, the fat mass and obesity‐associated protein; METTL3, methyltransferase‐like 3; METTL14, methyltransferase‐like 14; VIRMA, vir‐like m^6^A methyltransferase associated; YTHDF1/2/3, YTH‐domain‐containing proteins. (YTHDF2 in HIP, *p = .024; YTHDF3 in NAc, **p = .009; YTHDF3 in HIP, *p = .024.) Multiple *t*‐test, *p < .05; **p < .01; ***p < .001; *p* ≥ .05, not significant. Data represent means ± SEM (*n* = 5–6 animals). **(d)** The relative mRNA expression levels of Mu‐opioid receptor (Oprm1), normalized to GAPDH as a reference. (*p* = .033 in PVT; *p* = .009 in NAc). Multiple *t*‐test. *p < .05; *p* ≥ .05, not significant. Data represent means ± SEM (*n* = 5 animals.)

Previous research suggests that m^6^A level is associated with external stimulation, including responses to environmental stimuli, learning, sensory experiences, or injury (Stewart et al., 2021; Weng et al., 2018; Widagdo et al., 2016). In this study, we first aimed to investigate whether acute morphine stimulation will affect m^6^A modification or not. To examine this, we analyzed the m^6^A abundance in total RNA from the PRL, PVT, NAc, and HIP brain regions following an acute injection of morphine (MOR, *n* = 5–6) or NaCl (NaCl, *n* = 5–6). Using the m^6^A dot blot assay, we observed no significant change in m^6^A abundance in these brain regions (Figure 2a and b).

To further confirm the effect of acute morphine stimulation, we assessed the mRNA levels of m^6^A‐related proteins via qPCR assay. Specifically, we measured the mRNA levels of m^6^A demethylases (ALKBH5, FTO), methylases (Mettl3, Mettl14, KIAA1429), and m^6^A recognition proteins (YTHDF1, YTHDF2, YTHDF3) in the aforementioned four brain regions following an acute injection of morphine (MOR, *n* = 5–6) or NaCl (NaCl, *n* = 5–6). Our findings revealed no significant changes in the mRNA levels of most of these m^6^A‐associated proteins in the brain regions mentioned above, except for a decrease of YTHDF2 and YTHDF3 in the HIP and a decrease of YTHDF3 in NAc. It is important to note that the HIP was not activated by a single dose of morphine (Figure 2c). It is well known that morphine binds primarily to mu‐opioid receptors (MORs). We then characterized the expression of Oprm1 (opioid receptor mu 1) after acute morphine injection. qPCR data revealed an increase of Oprm1 in PVT and a decrease in NAc (Figure 2d), consistent with the key functional roles of these two regions in opioid dependence (Zhu et al., 2016).

Taken together, our results indicate that there were no significant changes in m^6^A abundance or the mRNA levels of m^6^A‐associated proteins in the brain regions activated by a single dose of morphine. This suggests that m^6^A modification may not be involved in the process of brain region activation by acute morphine.

### 3.3 Chronic morphine treatment and CPP confirmation

**FIGURE 3:**
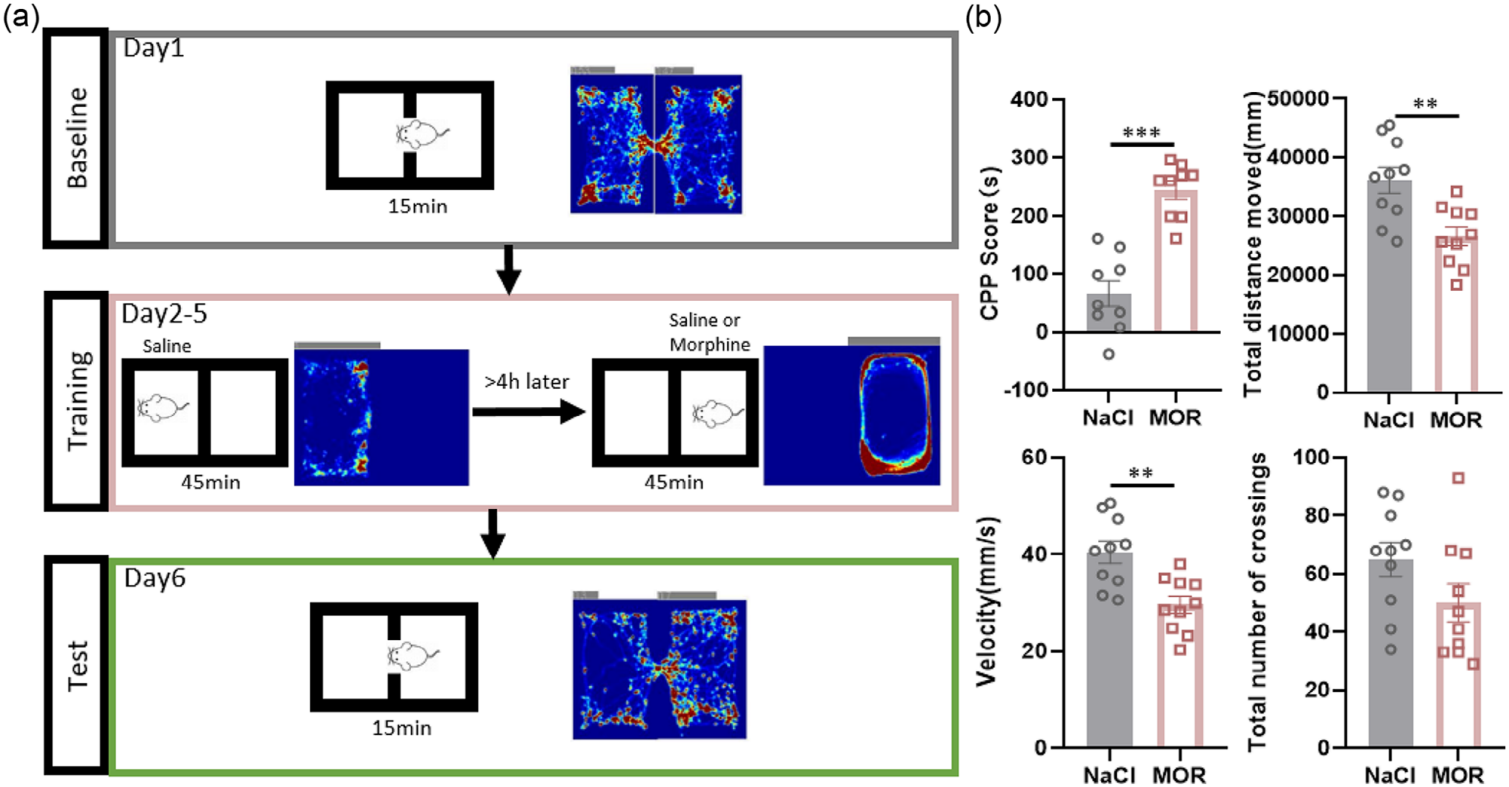
Chronic morphine treatment induced CPP model. (a) Schematic representation of the construction of the chronic morphine administration model. Baseline period (gray): to determine baseline preferences of mice; Training period (red): implementation of the continuous morphine exposure training regimen in a specific environment; Test period (green): assessment of the rewarding effect of morphine. **(b)** The extent of morphine preference (CPP score), total distance traveled, movement speed (Velocity), and the count of transitions between the two chambers (total number of crossings) were measured in both continuously morphine‐exposed (MOR) and nonexposed (NaCl) mice, during the test period. (***p = .0001 in CPP score, **p = .0026 in total distance moved, **p = .0015 in Velocity, *p* = .1037 in total number of crossings.) Unpaired *t*‐test. *p < .05; **p < .01; ***p < .001; *p* ≥ .05, not significant. Data represent means ± SEM (*n* = 9 animals).

The results in the m^6^A dot blot assay indicate that acute morphine injection did not induce significant changes in m^6^A levels (Figure 2a–c), suggesting that the overall abundance of m^6^A does not respond to the acute morphine stimulus. The role of m^6^A in chronic morphine exposure still requires investigation. Next, we want to investigate whether m^6^A modification plays a role in chronic morphine‐induced reward‐related behaviors. We established a mouse model of chronic morphine administration (Figure 3a). After the completion of the modeling process, we conducted conditioned place preference (CPP) testing to confirm the effect of chronic morphine treatment. During the CPP testing, mice that received morphine (MOR, *n* = 9) for four consecutive days exhibited a significant preference for staying in the morphine administration box compared to the NaCl injection box, as evidenced by higher CPP scores. But similar differences were not observed in the NaCl group (NaCl, *n* = 9) (Figure 3b). Notably, during the training phase, morphine administration induced increased locomotor activity, whereas NaCl administration did not (Figure 3a). During the test phase, the chronic morphine‐treated mice displayed a tendency to stay in the morphine administration box, resulting in a lower traveled distance, reduced speed, and fewer crossings between the morphine administration box and the NaCl injection box compared to the NaCl group (Figure 3b).

The successful establishment of conditioned preference in the morphine group was indicated by the observed behavioral changes in the CPP test. Next, we proceed with the further analysis of m^6^A modification in the mice exhibiting reward‐related behaviors induced by chronic morphine.

### 3.4 m^6^A level and m^6^A‐associated factors expression in chronic morphine‐treated mice

**FIGURE 4:**
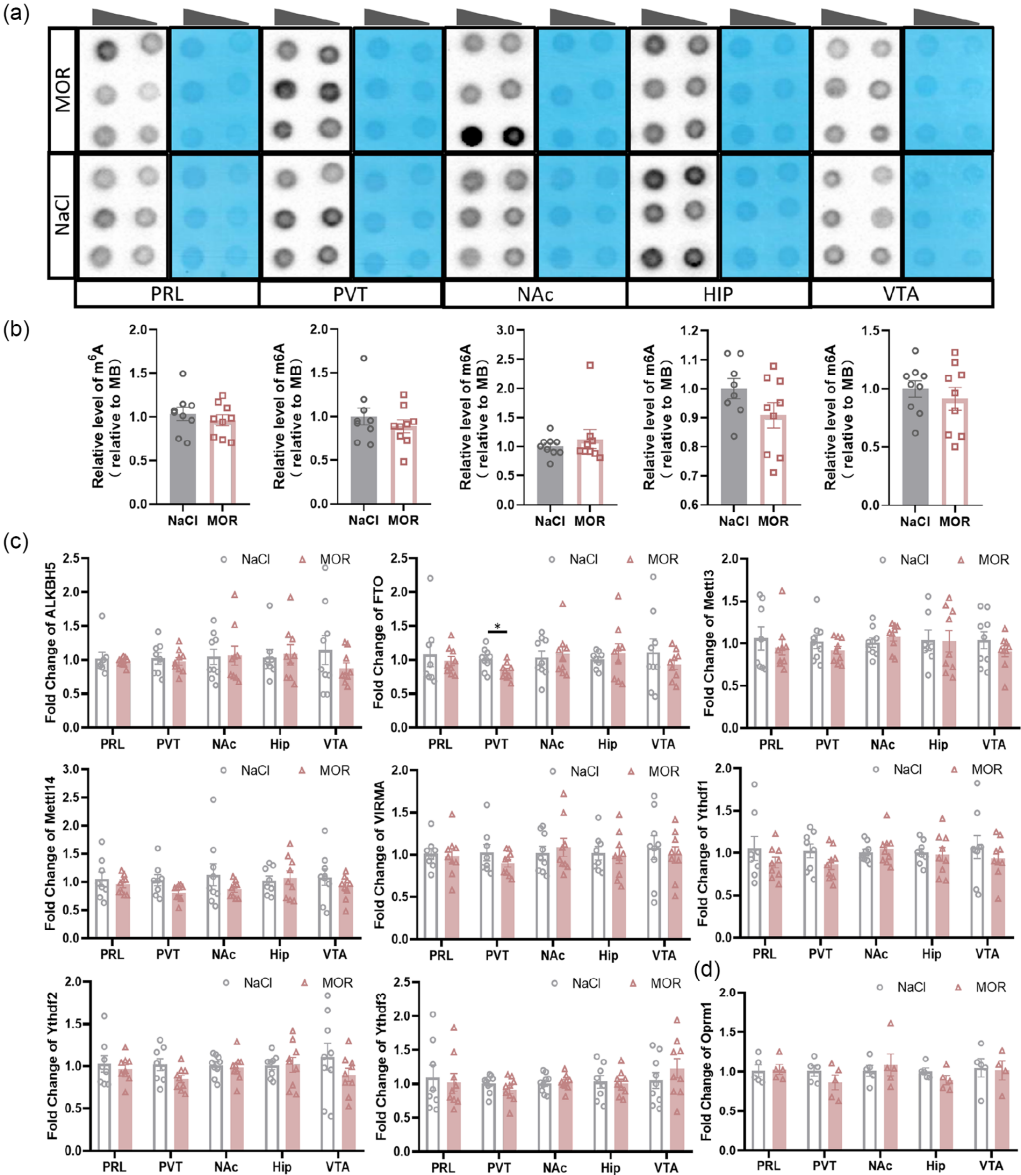
m^6^A modification landscape after chronic morphine exposure. (a) The level of m^6^A in total RNA was assessed using m^6^A dot blot in the PRL, PVT, NAc, HIP, and VTA regions of mice with morphine exposure (MOR, *n* = 8–9) and nonexposure (NaCl, *n* = 8–9). RNA samples were diluted to 150 ng, 75 ng for the analysis. **(b)** Quantification of m^6^A dot blot in the PRL, PVT, NAc, HIP, and VTA (*n* = 8–9), with methylene blue staining serving as the loading control. (*p* = .4501 in PRL; *p* = .3862 in PVT; *p* = .4692 in NAc; *p* = .1313 in HIP; *p* = .5117 in VTA.) *p* ≥ .05, not significant. Multiple *t*‐test. Data represent means ± SEM (*n* = 8–9 animals). **(c)** The relative mRNA expression levels of m^6^A‐related proteins, normalized to GAPDH as a reference, including ALKBH5, FTO, METTL3, METTL14, VIRMA, YTHDF1/2/3. (FTO in PVT, *p = .02.) Multiple *t*‐test. *p < .05; *p* ≥ .05, not significant. Data represent means ± SEM (*n* = 5–6 animals). **(d)** The relative mRNA expression levels of Mu‐opioid receptor (Oprm1), normalized to GAPDH as a reference. *p* ≥ .05, not significant. Multiple *t*‐test. Data represent means ± SEM (*n* = 4–5 animals).

It is well known that morphine abuse activates the dopaminergic system and affects synaptic plasticity by altering dendritic spines (Cahill et al., 2014; Tjon et al., 1994). In a previous study, we identified the crucial role of m^6^A in synaptic plasticity within the adult nervous system (Shi et al., 2018). Additionally, m^6^A has been implicated in reward behavior through the dopaminergic pathway (Hess et al., 2013; Sevgi et al., 2015; Sobczyk‐Kopciol et al., 2011). Thus, we speculated that m^6^A may play a role in the process of morphine‐induced reward‐related behaviors formation. Whereas we found no significant differences in the abundances of m^6^A in reward‐related brain regions (PRL, PVT, NAc, HIP, and VTA) between the chronic morphine‐treated mice (MOR, *n* = 8–9) and the control mice (NaCl, *n* = 8–9) (Figure 4a and b). Moreover, the mRNA levels of m^6^A‐associated proteins (MOR, *n* = 8–9; NaCl, *n* = 8–9) did not exhibit significant changes in the aforementioned brain regions, except for a slight decrease in FTO expression in PVT (Figure 4c). The effects of chronic morphine treatment on Oprm1 expression in these brain regions were also examined. It is widely accepted that long‐term morphine treatment did not alter mu‐opioid receptor mRNA levels (Brodsky et al., 1995). Consistent with this, we did not observe any changes in Oprm1 mRNA levels in all these regions after chronic morphine treatment (Figure 4d). These findings suggest that m^6^A modification may not play a primary role in the regulation of morphine‐induced reward‐related behaviors in mice.

Taken together, these results showed that m^6^A level and the expression of most m^6^A‐associated factors were not affected in key brain regions involved in reward and morphine response after both acute or chronic morphine treatment, indicating m^6^A modification may not participate in brain response to morphine exposure.

## 4 DISCUSSION

The effects of morphine on histone modifications and DNA methylation have been relatively well‐studied (Barrow et al., 2017; Chorbov et al., 2011; Doehring et al., 2013; Hwang et al., 2007; Mashayekhi et al., 2012; Wei et al., 2016), but there are limited researches on RNA methylation, especially m^6^A methylation. In this study, we investigated the overall level of m^6^A methylation in multiple brain regions during the acute stimulation phase of morphine, and after chronic morphine treatment. Interestingly, we found that the overall level of m^6^A methylation was not significantly changed in the brain regions that were activated or not activated during the acute stimulation of morphine. Moreover, the transcription levels of proteins related to m^6^A modification were also unchanged. Additionally, our results showed that the HIP, a brain region linked to learning, memory, and drug reward, exhibited no obvious of c‐fos activation following acute morphine stimulation. A comparable phenomenon was reported in a study exploring morphine‐induced transitions in brain states. The researchers discovered that c‐fos in HIP is not activated after the initial administration of morphine, but tends to be activated during morphine reinstatement after 24 h or four weeks of chronic morphine exposure (Brynildsen et al., 2020b). This suggests that hippocampal cell activation is intricately associated with morphine‐dependent states. Similarly, after the CPP test confirmed the formation of morphine‐induced preference, we found no changes in m^6^A levels in several key brain regions associated with reward in mice exposed to repeated morphine. Additionally, the transcription levels of proteins related to m^6^A modification were not significantly altered either. These results suggest that there is no significant change in the overall m^6^A methylation modification level during both acute and chronic morphine treatment in several key brain regions associated with drug reward, such as PRL, PVT, NAc, and HIP.

Our results show that the overall m^6^A modification is not directly altered in the morphine exposure model and there are no significant brain region‐specific differences. However, it is essential to consider that even when the overall level of modification is not affected, specific cell subsets or genes related to biological functions may still undergo specific modifications (Hess et al., 2013; Imperio et al., 2018). And we could not rule out the possibility that m^6^A modification may still play a role in the process of morphine‐induced rewarding effects. This possibility should not be discounted. For instance, it has been discovered that the m^6^A demethylase FTO may regulate cocaine responses specifically in dopaminergic neurons (Hess et al., 2013). The stress regulation of m^6^A is brain region specific (Engel et al., 2018) and specific regulation of various brain regions is primarily carried out by distinct functional cells. The distribution of m^6^A methylase in cells varies among different cell lines (Knuckles et al., 2017; Shi et al., 2019). These results suggest that m^6^A modifications may have cell type‐specific or transcription‐specific effects that could not be captured when examining the overall level.

In summary, this study suggests that the overall m^6^A modification level in different brain regions is not affected after different morphine exposure states. However, the possibility of specific modification effects on specific cell types or transcription‐specific loci cannot be ruled out. Further verification and investigation into cell‐specific or locus‐specific m^6^A modifications are necessary to gain a comprehensive understanding of the role of m^6^A in the context of morphine exposure and reward. This will help us unravel the complexities of epigenetic regulation in opioid exposure and contribute to the development of more targeted therapeutic approaches.

## AUTHOR CONTRIBUTIONS

**Xiaoli Wu**: Methodology; data curation; validation; formal analysis; writing—original draft. **Cuiting Wu**: Methodology; validation. **Tao Zhou**: Funding acquisition; project administration; supervision; writing—review and editing; writing—original draft.

## CONFLICT OF INTEREST STATEMENT

The authors declare no conflict of interest.

### PEER REVIEW

The peer review history for this article is available at https://publons.com/publon/10.1002/brb3.3350.

## Data Availability

The data that support the findings of this study are available from the corresponding author upon reasonable request.
